# LPMO-supported saccharification of biomass: effects of continuous aeration of reaction mixtures with variable fractions of water-insoluble solids and cellulolytic enzymes

**DOI:** 10.1186/s13068-023-02407-y

**Published:** 2023-10-21

**Authors:** Chaojun Tang, Madhavi Latha Gandla, Leif J. Jönsson

**Affiliations:** Department of Chemistry, Umeå University, 901 87 Umeå, Sweden

**Keywords:** Biodegradation, Lignocellulose, Enzyme, Biochemical conversion, Enzymatic saccharification, High substrate loading, Lytic polysaccharide monooxygenase, LPMO

## Abstract

**Background:**

High substrate concentrations and high sugar yields are important aspects of enzymatic saccharification of lignocellulosic substrates. The benefit of supporting the catalytic action of lytic polysaccharide monooxygenase (LPMO) through continuous aeration of slurries of pretreated softwood was weighed against problems associated with increasing substrate content (quantitated as WIS, water-insoluble solids, in the range 12.5–17.5%), and was compared to the beneficial effect on the saccharification reaction achieved by increasing the enzyme preparation (Cellic CTec3) loadings. Aerated reactions were compared to reactions supplied with N_2_ to assess the contribution of LPMO to the saccharification reactions. Analysis using ^13^C NMR spectroscopy, XRD, Simons’ staining, BET analysis, and SEM analysis was used to gain further insights into the effects of the cellulolytic enzymes on the substrate under different reaction conditions.

**Results:**

Although glucose production after 72 h was higher at 17.5% WIS than at 12.5% WIS, glucan conversion decreased with 24% (air) and 17% (N_2_). Compared to reactions with N_2_, the average increases in glucose production for aerated reactions were 91% (12.5% WIS), 70% (15.0% WIS), and 67% (17.5% WIS). Improvements in glucan conversion through aeration were larger (55–86%) than the negative effects of increasing WIS content. For reactions with 12.5% WIS, increased enzyme dosage with 50% improved glucan conversion with 25–30% for air and N_2_, whereas improvements with double enzyme dosage were 30% (N_2_) and 39% (air). Structural analyses of the solid fractions revealed that the enzymatic reaction, particularly with aeration, created increased surface area (BET analysis), increased disorder (SEM analysis), decreased crystallinity (XRD), and increased dye adsorption based on the cellulose content (Simons' staining).

**Conclusions:**

The gains in glucan conversion with aeration were larger than the decreases observed due to increased substrate content, resulting in higher glucan conversion when using aeration at the highest WIS value than when using N_2_ at the lowest WIS value. The increase in glucan conversion with double enzyme preparation dosage was smaller than the increase achieved with aeration. The results demonstrate the potential in using proper aeration to exploit the inherent capacity of LPMO in enzymatic saccharification of lignocellulosic substrates and provide detailed information about the characteristics of the substrate after interaction with cellulolytic enzymes.

## Background

The transition from an oil-based to a bio-based economy has received extensive attention during the last decades, primarily due to its potential with respect to addressing climate change and energy security [1]. Enzymatic deconstruction of lignocellulosic biomass to sugars is one of the main approaches to produce biofuels, such as cellulosic ethanol, and other bio-based chemicals [2, 3]. However, relatively high capital and operation expenditures contribute to making bio-based products expensive [4, 5]. Thus, technological advances are highly needed to make bio-based commodities more competitive and better suited to contribute to the bioeconomy.

Main steps in biochemical conversion of lignocellulosic biomass include pretreatment, enzymatic saccharification of cellulose, microbial fermentation of sugars, and valorization of lignin [6, 7]. Enzymatic saccharification of pretreated biomass at high-solids loadings has potential to improve the economic feasibility of the process [8, 9]. The process can be considered to be 'high-solids’ when the dry-matter content is ≥ 15% (w/w) [10]. Enzymatic saccharification performed at high-solids loadings offers advantages such as decreased capital and operation expenditures, lower energy demand and water input, and lowering wastewater generation and cost of disposal [11]. However, enzymatic saccharification with high-solids loadings has a drawback: the sugar yields decrease when solids loadings increase, which is referred to as the ‘high-solids effect’ [8]. Water constraints and inhibition of cellulolytic enzymes are factors that contribute to the high-solids effect [12]. Low fractions of free water cause high viscosity that results in poor mixing and limitations with respect to mass and heat transfer. This reduces the efficiency of the enzymes during the early stage of saccharification, known as the liquefaction stage [13]. Performing the saccharification with efficient enzymes and using optimal reaction conditions facilitates the liquefaction stage. The problem with poor mass transfer in reactions with high-solids loadings is then counteracted through rapid conversion of lignocellulosic slurries to more flowable mixtures [14]. Enzymes with high tolerance to end-product inhibition are also advantageous [15, 16].

Lytic polysaccharide monooxygenase (LPMO) has potential to improve enzymatic saccharification of cellulose through its ability to catalyze oxidative cleavage of glycosidic bonds and create more targets for hydrolytic enzymes [17, 18]. Whereas conventional enzymes used for saccharification of cellulose (such as cellobiohydrolase, endoglucanase, and β-glucosidase) are hydrolases, LPMO is an oxidoreductase. It requires an oxidant, such as molecular oxygen, as well as a reductant for activity [17, 18]. There are a few studies involving LPMO and reactions performed using high-solids loadings. In a comparison of different commercial cellulolytic enzyme preparations (Cellic CTec2 and Celluclast 1.5 L/Novozym 188), Cannella et al. [19] studied enzymatic saccharification of hydrothermally pretreated wheat straw using 30% WIS. Using Cellic CTec2, they found oxidized cellulose degradation products (gluconic acid, cellobionic acid) that were attributed to LPMO activity. Hu et al. [20] investigated how addition of accessory enzymes, xylanase and AA9 (LPMO), promoted enzymatic saccharification of steam-pretreated poplar and corn stover using 2%, 10%, and 20% (w/v) solids loadings. They found that both xylanase and LPMO improved saccharification, and that the effect of xylanase was dependent on the xylan content. Da Silva et al. [16] compared a laboratory-made enzyme blend with commercial enzyme preparations (Celluclast 1.5L/Novozym 188, Cellic CTec2, and Cellic CTec2/HTec2) using a solids content of 5%, 15%, or 20% in enzymatic saccharification of hydrothermally pretreated sugarcane bagasse. The glucose yields at 5% solids content were similar for all enzyme preparations, whereas Cellic CTec2 and Cellic CTec2/HTec2 performed better at 20% solids content. It is a possibility that the presence of LPMO in Cellic CTec2 and Cellic CTec2/HTec2 contributed to better performance at higher solids loading [16].

As the catalytic action of LPMO requires both an oxidant, such as molecular oxygen from air, and a reductant, such as lignin in the solid phase or lignin-degradation products in the liquid phase [21], it is often difficult to know if the LPMO that is present in modern enzyme preparations, such as Cellic CTec2 and Cellic CTec3, is efficiently utilized in saccharification reactions or not. Comparison of reactions that are continuously aerated with reactions that are supplied with N_2_ is a way to achieve a better understanding of the potential of LPMO to contribute to the saccharification reaction [21]. This approach was, however, not used in previous studies of using LPMO in reactions with high-solids loadings.

Factors such as increased enzyme dosage and utilization of conditions that permit the catalytic action of LPMO facilitate enzymatic saccharification, whereas using high-solids loadings make it more challenging. It is a critical question how large these positive and negative effects are in relation to each other. In the current investigation that question was addressed by investigating (*i*) the positive effects of LPMO using a modern commercial enzyme preparation (Cellic CTec3) in reactions with continuous supply of air or N_2_, (*ii*) the negative effects of increasing the content of WIS (water-insoluble solids) in the reaction mixture from 12.5% up to 17.5%, and (*iii*) the positive effects of increasing the enzyme dosage. Apart from that, the investigation also differs from previous work on LPMO and high-solids loadings by the use of pretreated softwood (Norway spruce) produced in a demonstration plant using continuous steam explosion with sulfur dioxide as catalyst, by using an experimental set-up allowing controlled gas addition with air and N_2_ to six parallel reaction mixtures, and by extensive analysis of both the liquid and the solid fractions before and after the reactions to understand how different reaction conditions affect the saccharification reactions. The techniques used to characterize the reaction mixtures included high-performance anion chromatography (HPAEC), compositional analysis through two-step treatment with sulfuric acid (TSSA), solid-state ^13^C NMR, X-ray diffraction (XRD), Simons’ staining, Brunauer–Emmett–Teller (BET) analysis, and scanning electron microscopy (SEM) analysis. Investigations in this area are important for achieving a better understanding of optimal technical design of processes based on biochemical conversion of lignocellulosic biomass.

## Results and discussion

### Analysis of pretreated biomass

The results of compositional analysis of the pretreated solids using TSSA are shown in Table 1. The pretreated solids contained 48.3% glucan and 48.7% total lignin (Klason lignin and ASL, i.e., acid-soluble lignin). Most of the lignin, 42.1%, was Klason lignin. The contents of other carbohydrates than glucan were very low (mannan and xylan) or below detection limits (galactan, arabinan). The total content of hemicellulosic carbohydrates (mannan, xylan, galactan, and arabinan) was  ≤ 0.4% (calculated from data in Table 1).

**Table 1:** Chemical composition of washed pretreated solids prior to enzymatic saccharification at 12.5% WIS with varied enzyme preparation loadings and after 72 h reactions with air or N_2_^a,b^

Constituent	PS	PS-Air			PS-N_2_		
		6%	9%	12%	6%	9%	12%
Glucan	48.3 (1.9)	31.9 (0.3)	26.6 (0.4)	26.9 (0.3)	41.0 (0.1)	39.2 (0.2)	36.8 (0.1)
Xylan	≤ 0.1	≤ 0.1	≤ 0.1	≤ 0.1	≤ 0.1	≤ 0.1	≤ 0.1
Mannan	≤ 0.3	≤ 0.3	≤ 0.3	≤ 0.3	≤ 0.3	≤ 0.3	≤ 0.3
Arabinan	ND	ND	ND	ND	ND	ND	ND
Galactan	ND	ND	ND	ND	ND	ND	ND
Klason lignin	42.1 (2.2)	54.2 (3.0)	56.4 (2.2)	55.4 (0.7)	46.3 (4.0)	46.7 (1.5)	49.6 (2.3)
ASL	6.6 (0.4)	5.8 (0.3)	5.9 (0.3)	6.1 (0.5)	5.7 (0.2)	5.8 (0.1)	5.4 (0.4)

^a^Determined using two-step treatment with sulfuric acid (TSSA). Values given as mass fraction in percent dry weight with standard deviation in parentheses. The values are averages of six measurements (duplicate samples of solid fractions and triplicate analyses of each sample)

^b^*ASL* acid-soluble lignin, *PS* pretreated solids, *ND* not detected, *WIS* water-insoluble solids

The fact that the total lignin content was about as high as the glucan content and the near absence of hemicellulosic carbohydrates shows that the pretreatment conditions had been very harsh. Norway spruce has a lignin content of 27.4% and a cellulose content of 41.7% [22]. As the total lignin content in the pretreated material was about as high as the glucan content, the lignin content must have been unusually high or the cellulose content unusually low, or both. The high Klason lignin content may be explained by the formation of pseudo-lignin, a Klason-lignin-positive aromatic substance formed through partial thermal decomposition of carbohydrates [7]. Pseudo-lignin is formed at high temperature and acidic conditions and is known to retard enzymatic saccharification [23]. Harsh pretreatment conditions can also lead to partial degradation of cellulose. That is not desirable, as there is a risk that monosaccharides formed during pretreatment are further degraded to undesired by-products, such as furan aldehydes and carboxylic acids [24].

### Enzymatic saccharification with varied WIS content

Data on glucose production from enzymatic saccharification with variable WIS content are shown in Table 2. The reactions with air always resulted in more glucose than the corresponding reactions with N_2_, and these differences were statistically significant (p ≤ 0.01). The increase in glucose production in reactions supported by aeration compared to corresponding reactions with N_2_ was: 34–112% after 24 h, 78–86% after 48 h, and 58–83% after 72 h. The average increase in glucose production in reactions supported by aeration after different time points was 91% for reactions with 12.5% WIS, 70% for reactions with 15.0% WIS, and 67% for reactions with 17.5% WIS. Thus, the average increase in glucose production was lower for reactions with higher WIS content, but the differences were not that great.

**Table 2:** Glucose yields from enzymatic saccharification of reaction mixtures with varied WIS and varied enzyme preparation loadings^a,b^

Reaction conditions		Produced glucose (g/L)^c,d^		
		24 h	48 h	72 h
WIS content^a^				
12.5%	Air	28.6 (2.7) ***	31.9 (1.0) ***	34.6 (2.2) ***
12.5%	N_2_	13.5 (1.7)	17.9 (1.9)	18.9 (0.7)
15.0%	Air	26.1 (2.2) ***	34.8 (2.3) ***	38.8 (1.8) ***
15.0%	N_2_	15.1 (3.2)	19.5 (2.6)	24.5 (1.9)
17.5%	Air	22.7 (4.0) ***	32.5 (0.7) ***	39.7 (2.2) ***
17.5%	N_2_	16.9 (0.7)	17.5 (1.9)	22.1 (3.1)
Enzyme preparation^b^				
6%	Air	25.3 (3.4) ***	33.1 (4.1) ***	37.1 (4.4) ***
6%	N_2_	15.0 (1.5)	18.3 (1.9)	19.9 (1.1)
9%	Air	32.3 (0.9) ***	37.9 (2.9) ***	43.6 (1.9) ***
9%	N_2_	19.3 (2.0)	21.8 (2.8)	25.9 (1.8)
12%	Air	37.0 (1.6) ***	44.2 (0.5) ***	47.3 (1.8) ***
12%	N_2_	19.7 (1.9)	22.0 (3.5)	25.9 (2.4)

^a^The series with varied content of water-insoluble solids (WIS) was performed with 6% enzyme preparation

^b^The series with varied enzyme loadings was performed with 12.5% WIS

^c^The table shows the glucose that was produced during enzymatic saccharification reactions, i.e., the glucose that was in the slurry as a result of the pretreatment has been deducted

^d^The values shown are averages of nine measurements (triplicate reactions and triplicate analyses of each reaction). Standard deviations in parentheses

Asterisks indicate significant differences (Student's t- test) between reactions with air and N_2_: ***p ≤ 0.01; ** 0.01 < p ≤ 0.05; * 0.05 < p ≤ 0.1

**Fig. 1:**
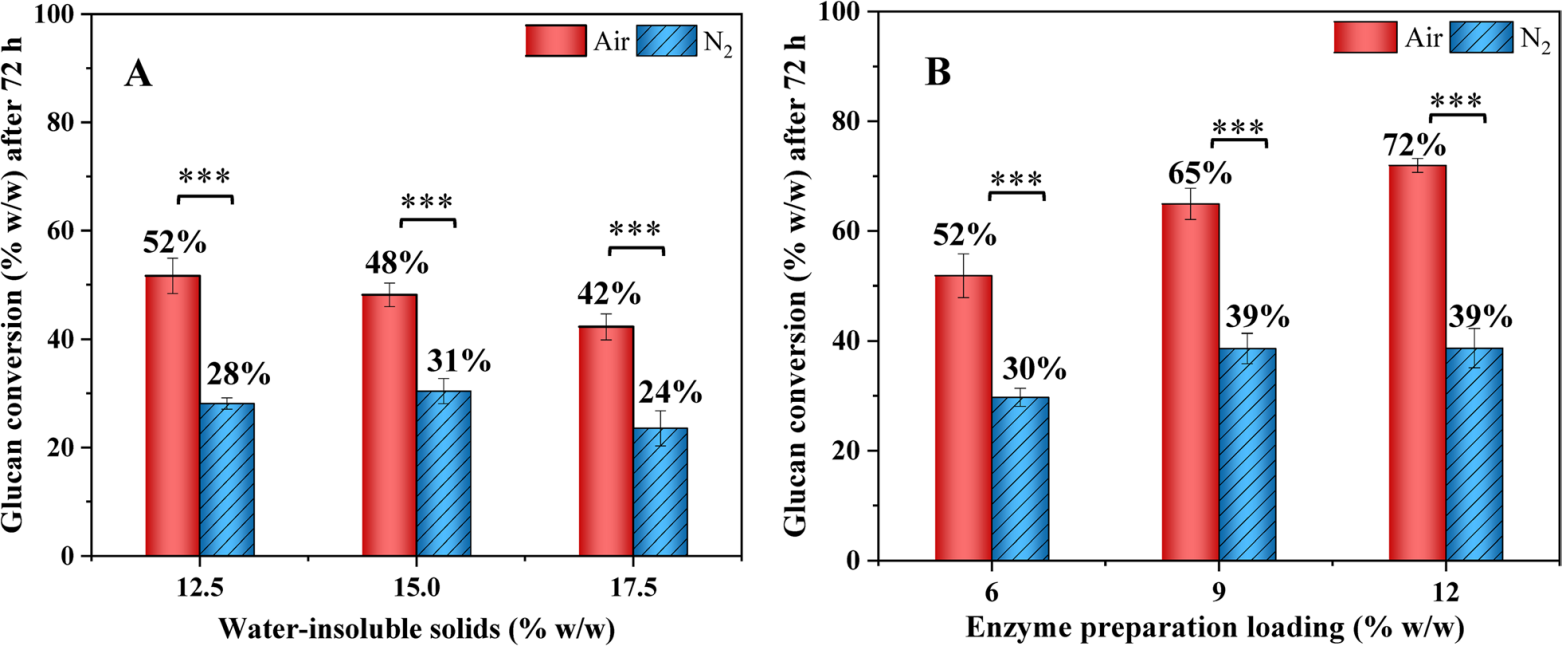
Glucan conversion in enzymatic saccharification of pretreated Norway spruce with **A** varying WIS and **B** varying enzyme preparation loading. Glucan conversion after 72 h of enzymatic saccharification was calculated according to Eq. (2) with a glucan content of 48.3% of pretreated solids. The values shown are averages of nine measurements (triplicate reactions and triplicate analyses of each reaction). Asterisks indicate significant differences (Student's t-test) between reactions with air and N_2_: ***p ≤ 0.01; **0.01 < p ≤ 0.05; * 0.05 < p ≤ 0.1

After 72 h, glucose production during enzymatic saccharification was slightly higher in reaction mixtures with higher WIS (Table 2). Aerated reactions with initial WIS content of 15.0–17.5% exhibited 39–40 g/L glucose production after 72 h, whereas the reaction with 12.5% initial WIS content exhibited only 35 g/L glucose production. Similarly, N_2_ reactions with initial WIS content of 15.0–17.5% exhibited 22–24 g/L glucose production, whereas the reaction with 12.5% initial WIS content exhibited only 19 g/L glucose production. However, the glucan conversion typically decreased with increasing WIS (Fig. 1A). In aerated reactions, the glucan conversion decreased from 52 to 42% when the WIS content increased from 12.5% to 17.5%. For reactions with N_2_, the corresponding decrease ranged from 28% (12.5% WIS) to 24% (17.5% WIS). The decrease in glucose conversion observed for aeration was statistically significant (p ≤ 0.05), whereas the decrease observed for reactions with N_2_ was not (p > 0.05)_._

It is noteworthy that the positive effects of aeration were much larger than the negative effects of increased WIS content. The increase in glucan conversion caused by aeration was in the range 55–86% whereas the decrease in glucan conversion caused by increasing the WIS content from 12.5% to 17.5% was in the range 17–24%. The glucan conversion with 17.5% initial WIS content and air (42%) was 50% higher than the glucan conversion with 12.5% initial WIS content and N_2_ (28%).

The experimental set-up resulted in clear differences both between reactions with varying WIS content and between reactions with N_2_ and air, although the former were rather small compared to the latter. This is relevant for process design, as the results show that aeration can compensate for the negative effects of high-solids loadings, at least up to 17.5% WIS. The increase in glucose production observed for aerated reactions can be attributed to the catalytic action of LPMO, as has been shown previously in experiments that included analysis of oxidation products resulting from the catalytic action of LPMO [21]. Other studies in which Cellic CTec3 has been used have also shown accumulation of LPMO oxidation products under aerobic reaction conditions [25]. Although LPMO is present also in reaction mixtures with N_2_, its catalytic effect is dependent on an oxidant, such as molecular oxygen, and it would not contribute to the saccharification reaction unless some residual molecular oxygen was present. The negative effect of high WIS content was expected, as the lack of free liquid water in a high-solids process results in poor mass transfer in the early hydrolysis stage [13]. Efficient enzymatic catalysis during the liquefaction stage would be expected to counteract poor mass transfer by improving the flow properties [14]. That was not obvious under the conditions used in the present study, as the negative effects of high-solids loadings were similar for both aerated reactions and reactions with N_2_. It is possible that such an effect would have been noticeable if an even higher WIS content than 17.5% had been included in the experimental set-up.

The glucose conversion levels in the experiments were rather modest. There are several reasons for that: Pretreatment using steam explosion under acidic conditions would typically not result in as good enzymatic digestibility as pretreatment using chemical pulping, for instance sulfite pulping, which was studied by Chylenski et al. [25]. For practical reasons, the saccharification reactions in the present study were interrupted after 72 h, before they were fully completed. The chemical analysis of the pretreated material showed that the pretreatment conditions had been harsh, which is typically associated with formation of pseudo-lignin, which affects enzymatic saccharification reactions negatively [23]. The pretreated solids had a high Klason lignin content, which would be problematic considering catalytically non-productive binding of cellulolytic enzymes to lignin and pseudo-lignin [26]. The reported glucan conversion values are based solely on glucose formation, but glucan can also be degraded to cellobiose and glucooligosaccharides, and, when LPMO is involved, to sugar acids (C1 and C4 oxidation products) [19, 21, 25]. The slurry included in the reaction mixtures would contain saccharides that contribute to feedback inhibition of cellulolytic enzymes [7, 12, 27]. Process configurations such as Simultaneous Saccharification and Fermentation (SSF) and Hybrid Hydrolysis and Fermentation (HHF) can be used to at least partially avoid feedback inhibition of cellulolytic enzymes by monosaccharides [7], but using these options would have made it more difficult to follow the enzymatic saccharification reactions. Furthermore, the liquid phase of pretreated biomass also contains other substances than saccharides, such as phenolics, that inhibit cellulolytic enzymes [7, 27].

### Saccharification with varied enzyme loading

Data on glucose production in enzymatic saccharification using different enzyme preparation loadings are shown in Table 2. The increase in glucose production in reactions supported by aeration compared to corresponding reactions with N_2_ was in the range 67–101%. Compared to reaction mixtures with 6% enzyme preparation, the glucose production in aerated reactions increased with 15–28% for 9% enzyme preparation and with 27–46% for 12% enzyme preparation. The largest increases (28% and 46%) were observed after 24 h. Compared to reaction mixtures with 6% enzyme preparation, the glucose production in reactions with N_2_ increased with 19–30% for 9% enzyme preparation and with 20–31% for 12% enzyme preparation. In contrast to aerated reactions, the largest increases were not necessarily observed after 24 h, and, most strikingly, the increase with 12% enzyme preparation compared to 9% was almost negligible (in the range 0–2% compared to 8–17% for the aerated series). A similar trend was observed for glucan conversion after 72 h (Fig. 1B). Glucan conversion for both air and N_2_ increased greatly when the enzyme preparation loading was increased from 6 to 9%, 25% for aerated reactions and 30% for reactions with N_2_. However, when the enzyme preparation loading was increased from 9% to 12% there was no difference between reactions with N_2_, which both showed 39% conversion. In contrast, aerated reactions exhibited 65% glucan conversion with 9% enzyme preparation and 72% conversion with 12% enzyme preparation. This corresponds to an 11% increase in glucose conversion for aerated reactions when the enzyme preparation dosage was increased from 9% to 12%. Expressed in a different way, an increase in enzyme preparation dosage with 50% (from 6% to 9%) improved glucan conversion with 30% for reactions with N_2_ and with 25% for reactions with air, whereas a doubling of enzyme preparation dosage (from 6% to 12%) improved glucan conversion with 30% for reactions with N_2_ and with 39% for reactions with air.

The result that increasing the enzyme preparation dosage from 9% to 12% led to an improvement for the aerated reactions but not for reactions with N_2_ is interesting from a mechanistic viewpoint. It suggests that at 12% enzyme preparation the reaction mixtures with N_2_ were saturated with hydrolytic enzymes and that air and LPMO activity was needed to create new substrate for hydrolytic enzymes in a synergistic manner.

Arantes and Saddler [28] suggested that the accessible surface area of cellulose was one of the most critical factors in limiting the extent of enzymatic hydrolysis of cellulose. LPMO has been reported to act in a complementary manner to cellulases by improving cellulose accessibility to the benefit of hydrolytic enzymes [29, 30]. The capability of LPMO to catalyze oxidative cleavage of highly ordered crystalline cellulose regions [30, 31] might enhance cellulose accessibility and promote the action of hydrolytic cellulose-degrading enzymes.

### Effects of enzymatic saccharification using varied enzyme loading and initial WIS content of 12.5% on the solid phase

Continuously controlled aeration in LPMO-supported enzymatic saccharification of cellulose resulted in promising improvements in sugar production in the experimental series with varied enzyme loading. To further investigate the role of aeration and LPMO activity in relation to substrate characteristics during the saccharification, the solid fractions before and after enzymatic saccharification were analyzed with regard to chemical composition, surface area, accessibility, crystallinity, and surface structure.

#### Chemical composition of pretreated solids

In agreement with the data in Table 2, reactions with N_2_ resulted in material with less glucan and more Klason lignin than in pretreated solids (PS), and reactions with air resulted in material with less glucan and more Klason lignin than reactions with N_2_ (Table 1). The ASL value was not much affected but was slightly lower in material from enzymatic reactions (5.4–6.1%) than in PS (6.6%).

The analysis of the chemical composition of the solid phase agrees with the glucose data in the sense that it shows that the effects of aeration were greater than the effects achieved by adding more enzyme to the reaction mixtures. The increase in Klason lignin content would merely reflect the decrease in glucan content. It is noteworthy that the ASL values did not follow the same pattern as the Klason lignin values, as they decreased rather than increased. It is a possibility that a part of the ASL was solubilized during the enzymatic reactions. This warrants more attention in future studies.

#### Brunauer–Emmett–Teller (BET) analysis

**Fig. 2:**
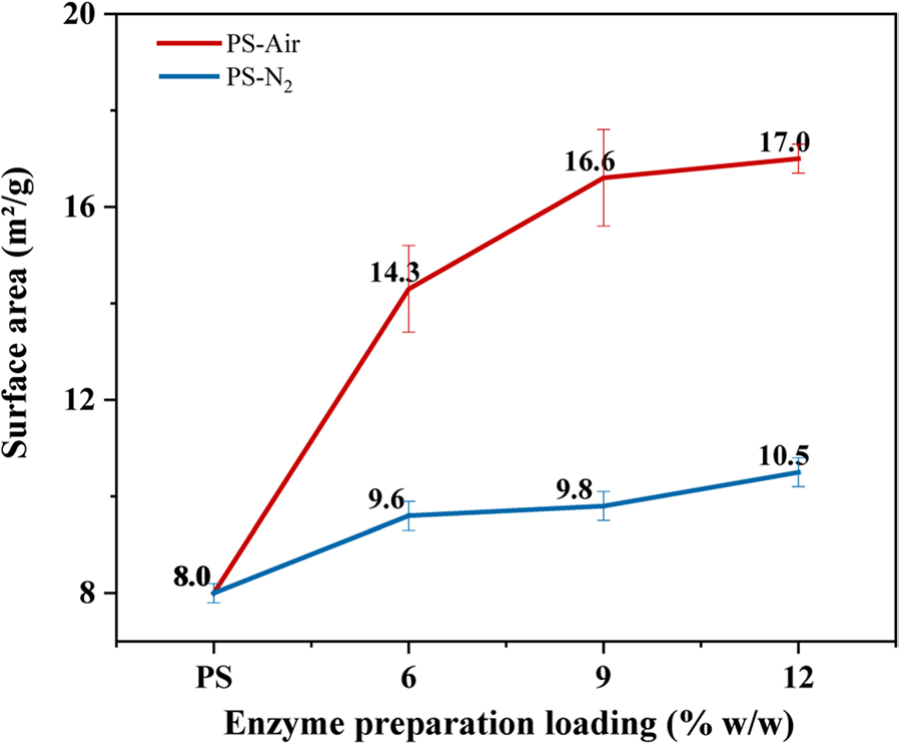
BET analysis of pretreated solids (PS) before and after 72 h of enzymatic saccharification at 12.5% WIS with varied enzyme preparation loadings in the presence of air or N_2_. The analysis was conducted in triplicate and error bars show standard deviations

Figure 2 shows the changes in surface area before and after enzymatic saccharification in the presence of air or N_2_ and using different enzyme preparation loadings, as determined using BET analysis. As the enzyme dosage increased, the surface area of the solid fraction consistently increased regardless of whether air or N_2_ was supplied. However, the increase was much larger for aerated reactions (from 8.0 to 14.3—17.0 m^2^/g) than for reactions with N_2_ (to 9.6–10.5 m^2^/g). Thus, the surface area increased with 79–112% in reactions with air and with 20–31% in reactions with N_2_.

The data points are too few to draw any clear conclusion about the relationship between the enzymatic saccharification reaction (measured as glucose production in g/L) and the surface area increase (measured as the increase in surface area in m^2^/g), but assuming a linear relationship results in a trendline with R^2^ 0.97 (data not shown). This suggests that the relationship was relatively close to linear in the range of conditions that were investigated. As BET analysis is based on the measurement of physical adsorption of nitrogen gas on solid surfaces, it reflects interactions between the solid phase and a small molecule, much smaller than the size of an enzyme. Studies of interactions with larger molecules, more similar to the size of enzymes, can instead be made using Simons' staining [32, 33].

#### Simons’ staining analysis

Simons’ staining has been used in the pulp and paper industry to assess the changes of fiber pore structures [34, 35]. The technique has also been used to evaluate the accessible surface area for enzymes involved in the deconstruction of cellulosic biomass [28, 32, 33, 36]. The Simons' stain includes two dyes, Direct Blue (DB), with a size of ~ 993 Da, and Direct Orange (DO), with a size of > 100 kDa [32]. Whereas DB is smaller than an enzyme, DO has a size that is similar to many cellulases [32]. The DO dye has been found to have higher affinity to cellulose than to hemicellulose and lignin [32].

**Fig. 3:**
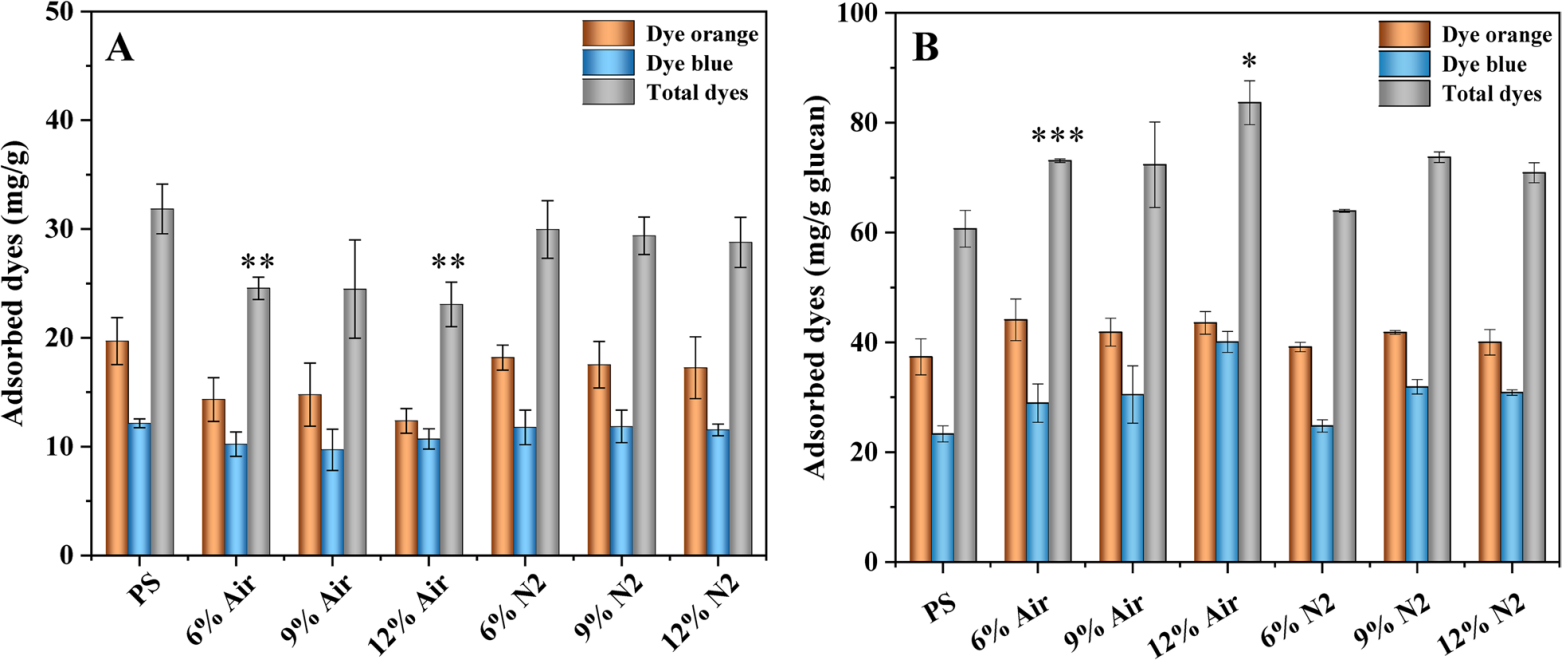
Adsorption of Simons' stain dyes on (**A**) pretreated solids and on (**B**) glucan before and after 72 h enzymatic saccharification at 12.5% WIS with varied enzyme preparation loadings in the presence of air or N_2_. The analysis was conducted in triplicate and error bars show standard deviations. Asterisks indicate significant differences (Student's t-test) between reactions with air and corresponding (same enzyme loading) reactions with N_2_: ***p ≤ 0.01, **0.01 < p ≤ 0.05, * 0.05 < p ≤ 0.1

Figure 3 shows the amount of adsorbed dye (separately and combined) per g solids (Fig. 3A) and per g glucan (Fig. 3B). PS showed the highest fraction of total adsorbed dye (31.8 mg/g), material from reactions with N_2_ showed intermediate values (28.8–30.0 mg/g), and material from reactions with air showed the lowest values (23.1–24.6 mg/g). DO followed the same trend as total adsorbed dye, and for DB material from reactions with air showed less adsorption than PS and material from reactions with N_2_. There was a weak but prevalent trend that reactions with more enzyme exhibited less adsorption than comparable reactions with less enzyme (Fig. 3A).

Total adsorbed dye on glucan content was calculated using TSSA data (Table 1) and the results are presented in Fig. 3B. Here, the adsorption of dyes increased rather than decreased after enzymatic saccharification. The material from reactions with air exhibited the highest fraction of total adsorbed dye (72.3–83.6 mg/g), PS exhibited the lowest value (60.7 mg/g), and material from reactions with N_2_ exhibited intermediate values (64.0–73.7 mg/g). Furthermore, statistical analysis revealed that the reactions with air and 6% or 12% enzyme preparation loadings resulted in statistically significantly higher adsorption of total dyes compared to the corresponding reactions with N_2_.

When the Simons' staining was evaluated as adsorbed dye per g solids (Fig. 3A), material from reactions with more extensive saccharification, i.e., from reactions with air, exhibited less adsorbed dye than material from reactions with no (PS) or less extensive (N_2_) saccharification. This can be explained by the fact that the cellulose content was reduced during the enzymatic saccharification reaction, as indicated by the glucan values in Table 1, and less adsorption, particularly of DO that has affinity for cellulose [32]. When the Simons' staining was instead evaluated as adsorbed dye per g glucan (Fig. 3B), the trend was the opposite, i.e., the reactions with air, which exhibited more extensive saccharification, showed more adsorbed dyes. This can be explained by DB having little or no affinity for cellulose, DO values being relatively steady due to the affinity of DO for cellulose [32], and total adsorbed dyes mainly reflecting the changes in DB values. It is particularly noteworthy that the DO values were higher for reactions with air than for PS and reactions with N_2_ (Fig. 3B), suggesting improved cellulose accessibility for cellulase enzymes.

#### X-ray diffraction analysis (XRD) and solid-state ^13^C-NMR

**Fig. 4:**
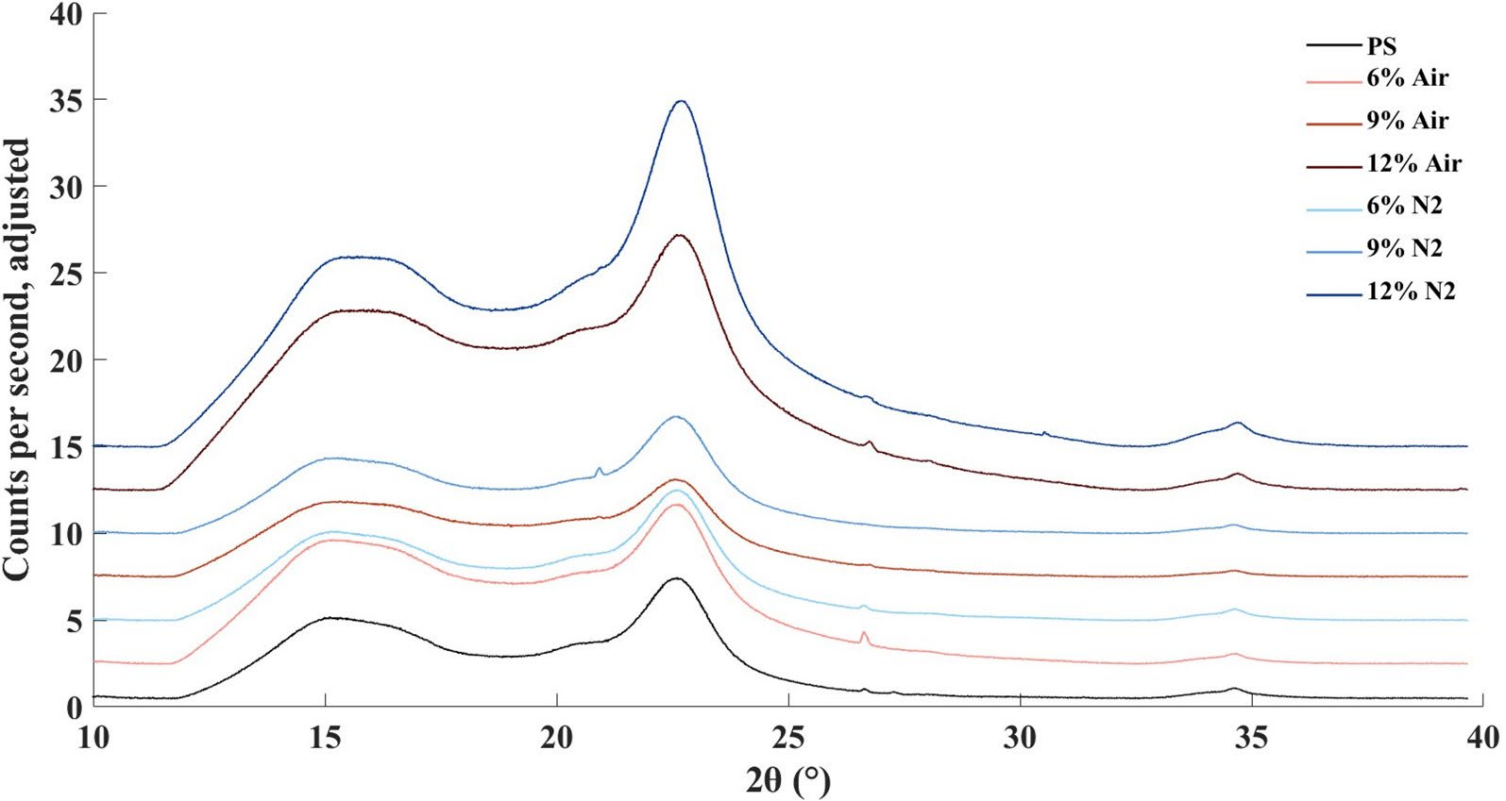
X-ray diffractograms from analysis of solid phases before and after enzymatic saccharification. The y-axis scale has been adjusted to facilitate comparisons

Figure 4 shows the XRD spectrum of PS and solid fractions after enzymatic reactions using different enzyme preparation loadings, and results including crystalline index (CrI%) are presented in Table 3. Material from LPMO-supported enzymatic reactions in the presence of air exhibited the lowest crystallinity index (44.4–49.9%) compared to N_2_ (59.3–62.6%) and PS (64.4%).

**Table 3:** Analysis of crystallinity of washed pretreated solids prior to enzymatic saccharification at 12.5% WIS with varied enzyme preparation loadings and after 72 h enzymatic reaction with air or N_2_^a^

	PS	PS-Air			PS-N_2_		
		6%	9%	12%	6%	9%	12%
XRD							
CrI %	64.4	49.9	47.5	44.4	59.3	62.6	60.3
2θ I_AM_ (°)	18.92	19.18	19.12	18.85	18.96	18.99	18.73
I_AM_ (CPS)	2.42	4.56	2.99	8.18	2.99	2.48	7.83
2θ I_200_ (°)	22.57	22.57	22.57	22.63	22.58	22.57	19.72
I_200_ (CPS)	6.81	9.09	5.70	14.7	7.51	6.63	22.66
^13^C NMR^b^							
Int.(89)	1307	961	865	865	1172	1145	1114
Int.(84)	716	544	497	496	626	626	606
Int.(89)/Int.(84)	1.83	1.77	1.74	1.74	1.85	1.83	1.84
CrI (%)	64.6	63.9	63.5	63.6	64.9	64.7	64.8

^a^*CPS* counts per second, *CrI* crystallinity index, *PS* pretreated solids, *NMR* nuclear magnetic resonance spectroscopy, *WIS* water-insoluble solids, *XRD* X-ray diffraction

^b^The relative intensities of the peaks at 89 ppm (crystallinity region) and 84 ppm (amorphous region) are listed

**Fig. 5:**
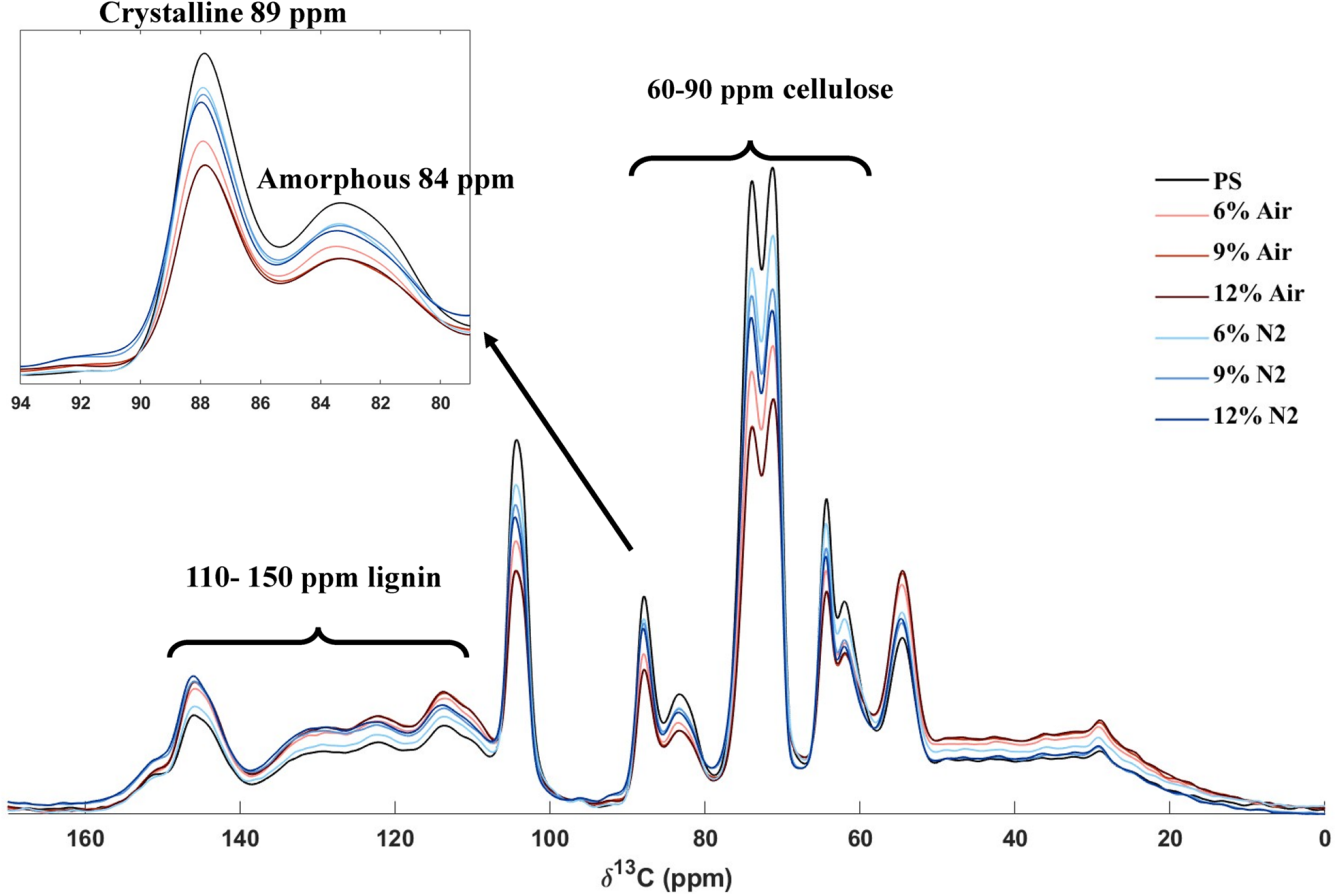
Solid-state^13^C NMR spectra of pretreated solids (PS) and solids after 72 h of enzymatic saccharification at varied enzyme preparation loadings in reactions with 12.5% WIS and with air or N_2_

The solid fractions were further analyzed using solid-state ^13^C-NMR. The spectra obtained after total normalization are shown in Fig. 5. The differences with regard to the relative amounts of cellulose and lignin are clearly visible, and agree with results from enzymatic saccharification (Table 2) and compositional analysis (Table 1). NMR can be used to estimate the crystallinity index, typically based on the peak at 89 ppm assigned to the C4 carbon in the ordered cellulose structure and the peak at 84 ppm assigned to the C4 carbon of disordered cellulose [37]. The differences in crystallinity index were not as clear as in the data from the XRD analysis, but agree in the sense that CrI for material from reactions with air (63.5–63.9%) was slightly lower than for PS and materials from reactions with N_2_ (64.6–64.9%) (Table 3).

The results from the XRD experiments were clear-cut and consistent with other results, whereas the results from the NMR experiments were more difficult to interpret. That the two methods gave somewhat different results is not entirely unexpected, as determination of crystallinity has been found to be method dependent and deviations in results obtained using different techniques are common [38]. Cellulose crystallinity has often been assumed to have a negative effect on cellulose digestibility [39, 40]. However, even if cellulose crystallinity has an effect in experiments with pure cellulosic substrates other studies indicate that it is not among the more important factors governing enzymatic saccharification of lignocellulosic substrates, at least not compared to factors such as hemicellulose content and cell wall architecture [41]. Nevertheless, the clear decrease in crystallinity observed for aerated reactions using XRD indicates that LPMO improved the conversion of crystalline cellulose.

#### Scanning electron microscopy

**Fig. 6:**
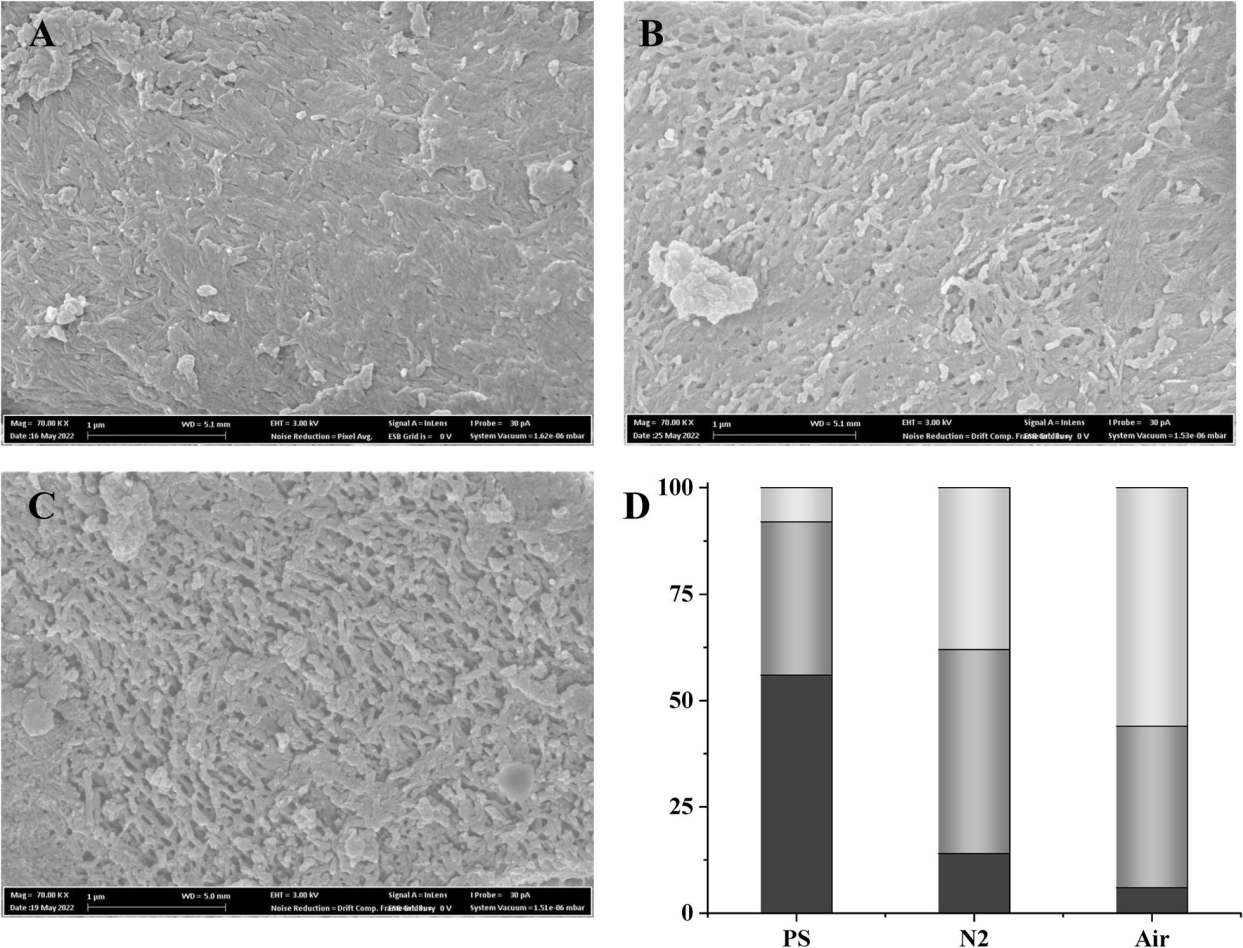
SEM micrographs of (**A**) pretreated solids (PS), and of solids after 72 h of enzymatic saccharification (12.5% WIS at 12% enzyme preparation loading) with **B** N_2_ or **C** air. PS exhibits a relatively ordered structure, N_2_ a less ordered structure, and air a highly disordered structure. The graph on the lower right side **D** shows analysis of 50 SEM images of each of PS, PS-N_2_, and PS-Air and the percentage of each type of structure. Black bars refer to an ordered structure, grey bars to a less ordered structure, and light grey bars to a disordered structure

Structural changes after LPMO-supported enzymatic saccharification in the presence of air and N_2_ are shown in Fig. 6. Pretreated solids prior to enzymatic hydrolysis exhibited a relatively ordered structure (Fig. 6A). Solids from reactions with N_2_ showed a less ordered structure (Fig. 6B), whereas solids from reactions with air showed a highly disordered structure (Fig. 6C). For statistic evaluation, surface structures were defined as ordered, less ordered, and disordered, and 50 randomly collected SEM images from each of PS, material from reactions with air, and material from reactions with N_2_ were evaluated (Fig. 6D). The result clearly showed that there were more ordered structures in PS (56%) than in materials from reactions with N_2_ (14%) or air (6%). Less ordered structures were most prevalent (48%) in material from reactions with N_2_, and disordered structures were most prevalent (56%) in material from reactions with air (Fig. 6D). The observations agree with the data in Tables 1 and 2.

SEM analysis has been widely used to observe structural changes of cell walls after using different pretreatment methods [42–44]. This investigation shows that SEM analysis is also a useful tool for both qualitative and quantitative (Fig. 6D) analysis of enzymatic saccharification reactions. Aerated reactions with LPMO were superior for creating a disordered cell wall structure, an effect that greatly enhanced the efficiency of the saccharification reactions.

## Conclusions

The potential benefit of continuous aeration in LPMO-supported enzymatic saccharification was investigated in experiments with softwood that was pretreated using continuous steam explosion with sulfur dioxide as a catalyst and using variable WIS content and enzyme loading in the reaction mixtures. Analysis of the substrate showed that the pretreatment had been harsh, as indicated by very low levels (≤ 0.4%) of residual hemicelluloses in the pretreated solids and also by relatively low glucan conversion levels. As no external reductant was added to the reaction mixtures, LPMO activity was dependent on lignin and lignin degradation products in the pretreated biomass. The results show that the positive effects of aeration, with molecular oxygen in the air enabling catalysis by LPMO, were larger than the negative effects of high-solids loadings in the interval 12.5–17.5% WIS content. Consequently, the glucan conversion with aeration at 17.5% WIS was larger than the glucan conversion with N_2_ at 12.5% WIS. Furthermore, doubling the enzyme loading was not as efficient in promoting glucan conversion as applying aeration. The general applicability of the findings needs to be further investigated using other feedstocks and milder pretreatment conditions, but the investigation clearly shows that there is a great potential in applying continuous aeration to promote LPMO activity in reactions with high-solids loadings.

## Methods

### Materials

Slurry from unbarked Norway spruce (*Picea abies*) pretreated using continuous steam explosion with sulfur dioxide as catalyst was prepared in the Biorefinery Demonstration Plant (BDP) (Örnsköldsvik, Sweden) by SEKAB E-Technology AB. The wood chips were impregnated with approx. 2% sulfur dioxide (based on dry weight of biomass) and treated at 205 °C for approx. 10 min. The pH of the resulting slurry was approx. 1.8.

### Determination of the content of water-insoluble solids (WIS)

20 g of slurry in a 50 mL Falcon tube was centrifuged at 9 000 rpm for 20 min at 4 °C (Centrifuge 5810 R, Eppendorf, Germany). The supernatant was decanted taking care to minimize the loss of insoluble solids. Approx. 20 mL ultra-pure water was added, and the solids were re-suspended by hand mixing and centrifuged. The process was repeated until the glucose concentration in the supernatant was below 0.05 g/L as measured using a glucometer (Accu-Chek Aviva, Roche Diagnostics GmBH, Mannheim, Germany). The washed solids were dried at 105 °C over night. Dried solids were kept in a desiccator to avoid uptake of moisture after drying and the weight was measured. The calculation of water-insoluble solids was done by using Eq. 1 from [45]:

1 $${\text{\% }}\,{\text{fraction}}\,{\text{of}}\,{\text{insoluble}}\,{\text{solids}}\,{ = }\,\frac{{{\text{oven - dry}}\,{\text{weight}}\,{\text{of}}\,{\text{washed}}\,{\text{solids}}\,{\text{(g)}}}}{{{\text{weight}}\,{\text{of}}\,{\text{wet}}\,{\text{slurry}}\,{\text{(g)}}}}\, \times \,{100}$$

The WIS content of the slurry (subsection "Materials") was determined to 19.4%.

### Enzymatic saccharification reactions with varied WIS content

Three series of enzymatic saccharification experiments (12.5%, 15.0%, and 17.5% WIS) were performed. The pH of the slurry was first adjusted to 5.2 by using a 10 M aqueous solution of sodium hydroxide. Different WIS levels were prepared by dilution with ultra-pure water. If not otherwise stated, reaction mixtures (70 g) contained pH-adjusted slurry (resulting in 12.5%, 15.0%, or 17.5% WIS), 6% (w/w, based on the mass of the liquid enzyme preparation and the mass of WIS) Cellic^®^ CTec3 enzyme preparation (Novozymes A/S, Bagsvaerd, Denmark), and 1% (w/w, based on the WIS content) antifoaming agent (Tween 80). A separate experiment using spruce slurry, Cellic CTec3, and triplicate reaction mixtures was performed to assure that inclusion of the antifoaming agent did not affect the enzymatic reaction (data not shown).

Reaction mixtures were prepared in gas wash bottles (125 mL) (Quickfit^®^ Drechsel bottles from Sigma-Aldrich, St Louis, MO, USA). The bottles were equipped with magnetic stirrer bars (cylindrical 25 × 6 mm) and six parallel bottles were positioned on a multipoint stirrer (Cimarec from Thermo Scientific, Waltham, MA, USA) immersed in a water bath to allow for temperature control. The stirring speed was 110 rpm. A continuous supply of air (three bottles) or N_2_ (99.996% purity) (three bottles) into the gas wash bottles was achieved using Polyamide 12 tubing (outer diameter 6 mm). For each gas supply, a gas distributor was used to divide the gas into three strands, and each gas strand was connected to a flow meter (Model F65, Porter Instrument Division, Parker Hannifin Corporation, Hatfield, PA, USA). The gas flow was kept at 0.2 vvm (based on flow rate and reaction mixture volume; i.e., gas volume per reaction mixture volume and min). Reaction mixtures connected to N_2_ were flushed with N_2_ for around 5 min before the addition of the enzyme preparation. Zero samples (0 h) were collected before adding enzyme. Incubation was conducted at 45 °C for 72 h, and samples (0.5 mL each) were withdrawn after 24 h, 48 h, and 72 h by using an automatic pipette. The monosaccharides produced in the reactions were analyzed using high-performance anion-exchange chromatography (HPAEC) (subsection "Analysis of monosaccharides using HPAEC").

### Saccharification reactions with varied enzyme loading

A series of enzymatic saccharification reactions with varying enzyme preparation loadings (6%, 9%, and 12%) was conducted using 12.5% WIS. The set-up of the experiment was otherwise the same as previously described (subsection "Enzymatic saccharification reactions with varied WIS content"). After enzymatic saccharification, the reaction mixtures were centrifuged to separate the solid and liquid phases. The solids were washed with ultra-pure water until no glucose was detected in the filtrate. Washed solids were air-dried and sieved using sieve shaker AS 200 (Retsch, Haan, Germany). The dry-matter contents of the washed and dried solids were > 96%, as determined using an HG63 moisture analyzer (Mettler-Toledo, Greifensee, Switzerland). After sieving, the fractions of solids with a size below 100 µm were collected for further analysis: PS, pretreated solids; PS-Air, solid fraction after 72 h enzymatic saccharification with air; PS-N_2_, solid fraction after 72 h enzymatic saccharification with N_2_.

### Analysis of monosaccharides using HPAEC

Monosaccharides were analyzed by using high-performance anion-exchange chromatography (HPAEC) with pulsed amperometric detection (PAD). All samples were diluted with ultra-pure water and were filtered through 0.20 µm nylon membrane filters (Merck Millipore Ltd., Cork, Ireland) before injecting into the system for separation. The separation (ICS-5000) was performed using a separation column, CarboPac PA1 (4 × 250 mm), coupled with a guard column, CarboPac PA1 (4 × 50 mm), and using an electrochemical detector (all from Dionex, Sunnyvale, CA, USA). The column oven was set at 30 °C. The column was equilibrated during 14 min by using a mixture consisting of 60% Solvent B (an aqueous solution of 300 mM sodium hydroxide) and 40% Solvent C (an aqueous solution of 200 mM sodium hydroxide and 170 mM sodium acetate). Elution of the samples was performed during 25 min using ultra-pure water (Solvent A) at a flow rate of 1 mL/min. Quantitation of monosaccharides was conducted using external calibration standards in the range 0.5–30 mg/L. Each sample was analyzed in triplicate. Chromeleon 7.1 software (Dionex) was used for data analysis.

Glucan conversion during enzymatic saccharification (Eq. 2) was calculated based on the amount of produced glucose in relation to the glucan content of the substrate:

2 $${\text{Glucan}}\,{\text{conversion}}\,{\text{(\% )}}\,{ = }\,\frac{{{\text{(C}}_{{\text{t}}} \, - \,{\text{C}}_{{0}} {)}\, \times \,{\text{V}}\, \times \,{0}{\text{.9 }}}}{{{\text{glucan content}}\, \times \,{\text{m }}}}\, \times \,{100}$$

In Eq. 2, "C_t_" is the glucose concentration (in g/L) in a sample taken during the enzymatic saccharification reaction, "C_0_" is the glucose concentration (in g/L) in the reaction mixture before the start of the enzymatic saccharification reaction, "V" is the volume of the reaction mixture (in L), "glucan content" is the fraction of glucan in the water-insoluble solids before the start of the enzymatic saccharification reaction, and "m" is the mass (in g) of water-insoluble solids in the reaction mixture before the start of the enzymatic saccharification reaction. The glucan content of the PS was obtained using data from the compositional analysis (subsection "Compositional analysis using two-step treatment with sulfuric acid"). Student’s t-test was used for statistical analysis of results.

### Analysis of solid fractions before and after enzymatic saccharification

#### Compositional analysis using two-step treatment with sulfuric acid

Two-step treatment with sulfuric acid (TSSA) was used to determine the contents of carbohydrates and lignin in the solid fractions according to the NREL/TP-510-42618 protocol [46] with some modifications. Instead of high-performance liquid chromatography (HPLC), HPAEC was used for determination of monosaccharides (subsection "Analysis of monosaccharides using HPAEC"). Acid-soluble lignin (ASL) was determined at λ 240 nm using a UV-1800 spectrophotometer (Shimadzu, Kyoto, Japan). Glass crucibles with integral glass sintered discs (Pyrex 2, porosity 10–16 µm) were used for gravimetrical determination of acid-insoluble lignin (Klason lignin). All analyses were conducted in triplicates.

#### Brunauer–Emmett–Teller (BET) analysis

BET analysis was used to assess the surface area of pretreated solids, prepared as described for the saccharification reactions, using a single-point BET procedure with a TriStar 3000 analyzer (Micromeritics, Atlanta, GA, USA). BET analysis is based on physical adsorption of gas molecules on a solid surface to measure its specific surface area [47]. Each sample consisted of 100 mg dried solids in a glass tube. Prior to analysis, degassing was performed using a SmartPrep Degasser (Micromeritics) to remove potential contaminants. Liquid nitrogen (~ 99.9996% purity) was utilized in the degassing step. Analysis was conducted in triplicate.

#### Simons’ staining analysis

Direct Orange (DO, Pontamine Fast Sky Orange 6RN) and Direct Blue (DB, Pontamine Fast Sky Blue 6BX) were obtained from Pylam Products (Garden City, NY, USA) and were used for the Simons’ staining analysis. A high molecular fraction of DO was prepared by using an Amicon^®^ stirred ultrafiltration apparatus (Millipore Corporation, Bedford, MA, USA) with a 100 kDa ultrafiltration membrane. A series of mixtures were prepared that consisted of 25 mg dry weight samples (prepared as described in subsection "Saccharification reactions with varied enzyme loading"), 0.25 mL phosphate-buffered saline solution, and different concentrations of the two dyes. The mixtures were incubated at 65 °C for 6 h. The procedure of Chandra and Saddler [32] was used with a slight modification of the incubation temperature (65 °C instead of 70 °C). The supernatant was collected and measured at 455 nm and 624 nm. Each sample was conducted in triplicate. The concentration of dyes in the supernatant was calculated based on Lambert–Beer’s law using Eqs. 3 and 4 [33]:

3 $${\text{A}}_{{{\text{455nm}}}} \,{ = }\,{\upvarepsilon }_{{\text{DO/455}}} \, \times \,{\text{L}}\, \times \,{\text{C}_{\text{O}}}\, + \,{\upvarepsilon }_{{\text{DB/455}}} \, \times \,{\text{L}}\, \times \,{\text{C}_{\text{B} }}$$

4 $${\text{A}}_{{{\text{624nm}}}} { }\, = \,{\upvarepsilon }_{{\text{DO/624}}} \, \times \,{\text{L}}\, \times \,{\text{C}_{\text{O}}}\, + \,{\upvarepsilon }_{{\text{DB/624}}} \, \times \,{\text{L}}\, \times \,{\text{C}_{\text{B} }}$$

In Eqs. 3 and 4, "A" refers to the absorption at 455 and 624 nm, respectively. The extinction coefficient (Direct Orange, ε_DO_; Direct Blue, ε_DB_) of the dyes at the respective wavelengths were (L g^−1^ cm^−1^): ε_DO/455_, 35.62; ε_DB/455_, 2.59; ε_DO/624_, 0.19; ε_DB/624_, 15.62. L is the path length (cm) of the microplates used [in this study 0.535 cm, using 96 well microplates from Sarstedt (Nümbrecht, Germany)].

#### X-ray diffraction (XRD)

The crystallinity index (CrI) of pretreated solids was determined using XRD. Diffractograms were collected with a D8 Advance instrument (Bruker-AXS, Karlsruhe, Germany) in θ-θ mode using Cu Kα1,2 radiation and a Våntec-1 detector. Samples were mounted on a low background Si sample holder. Continuous scans were applied within the 2θ range of 10°–40°. The data was collected until diffractograms appeared smooth. First, background removal of collected diffractograms was performed. The points for I_AM_ and I_002_ were identified with support of a 4th degree polynomial fit as these are required for calculation of CrI. CrI was calculated based on the intensity ratio between I_AM_ and I_200_ expressed as counts per seconds, using Eq. 5 [38]:

5 $${\text{CrI}}\,(\%) =\frac{\text{(I}_{002} - {\text{I}}_{\text{AM}})}{{\text{I}_{002}}} \times {100}$$

In Eq. 5, "I_AM_" represents the intensity of diffraction of non-crystalline material, which is defined as the minimum of the valley between the main cellulose peaks (specifically the range 17.8°–19.8°). "I_002_" refers to the maximum intensity of the peak that corresponds to the plane with the Miller indices 002 at a 2θ angle of 22°–24°.

#### Scanning electron microscopy (SEM)

Pretreated solids were dispersed onto carbon tape-mounted on an aluminum stub and sputter-coated with 10 nm platinum (Quorum Q150T ES). The morphology of the pretreated solids before and after 72 h enzymatic hydrolysis both with air and with N_2_ (PS, PS-Air and PS-N_2_ at 12% enzyme preparation loading) was investigated using field-emission scanning electron microscopy (FESEM, Carl Zeiss Merlin GmbH, Germany) using an in-lens secondary electron detector, accelerating voltage 3 kV, and probe current 30 pA. Analysis was conducted at the Umeå Core Facility Electron Microscopy (UCEM) of the Chemical-Biological Centre (KBC) (Umeå, Sweden). Fifty images of each sample were collected and classified into three types of structures: ordered, less ordered and disordered. The proportions of each type of structure in the 50-image sample were calculated.

#### Solid-state ^13^C-NMR

Solid-state ^13^C-NMR spectra were recorded with cross-polarization and magic angle spinning (CP-MAS) on an 11.7 T (500 MHz 1H) Bruker Avance III spectrometer equipped with a 4 mm MAS probe. Approximately 70 mg of each sample was loaded into 4 mm ZrO_2_ rotors. A 1 ms ramped contact pulse was used and 8 192 scans were added for each sample. A ^1^H decoupling frequency for 83 kHz was applied during the 23 ms acquisition time and the relaxation delay was set to 2 s. All spectra were calibrated using adamantane as an external chemical shift reference. Total normalization of spectra to a constant sum was performed in Matlab r2020a (Mathworks Inc.). The relative intensities of peak 89 ppm (crystallinity region of cellulose) and peak 84 ppm (amorphous region of cellulose) were summarized and used for the calculation of crystallinity index (CrI) according to Eq. 6:

6 $${\text{CrI}}\,{\text{(\% )}}\,{ = }\,\frac{{{\text{Int}}{.}\,{(89)}}}{{{\text{Int}}{.}\,{(89)}\, + \,{\text{Int}}{.}\,{(84) }}}\, \times \,{100}$$

## Data Availability

The datasets used and/or analyzed during the current study are available from the corresponding author on reasonable request.
